# Temozolomide in Glioblastoma Therapy: Role of Apoptosis, Senescence and Autophagy. Comment on Strobel et al., Temozolomide and Other Alkylating Agents in Glioblastoma Therapy. *Biomedicines* 2019, *7*, 69

**DOI:** 10.3390/biomedicines7040090

**Published:** 2019-11-11

**Authors:** Bernd Kaina

**Affiliations:** Institute of Toxicology, University Medical Center Mainz, Obere Zahlbacher Strasse 65, D-55130 Mainz, Germany; kaina@uni-mainz.de

**Keywords:** Temozolomide, alkylating agents, DNA repair, apoptosis, senescence

## Abstract

Temozolomide, a DNA methylating drug, is currently being used first-line in glioblastoma therapy. Although the mode of action of this so-called S_N_1 alkylating agent is well described, including the types of induced DNA damage triggering the DNA damage response and survival and death pathways, some researchers expressed doubt that data mostly obtained by in vitro models can be translated into the in vivo situation. In experimental settings, high doses of the agent are often used, which are likely to activate responses triggered by base N-alkylations instead of *O*^6^-methylguanine (*O*^6^MeG), which is the primary cytotoxic lesion induced by low doses of temozolomide and other methylating drugs in *O*^6^-methylguanine-DNA methyltransferase (MGMT) repair incompetent cells. However, numerous studies provided compelling evidence that *O*^6^MeG is not only a mutagenic, but also a powerful toxic lesion inducing DNA double-strand breaks, apoptosis, autophagy and cellular senescence. MGMT, repairing the lesion through methyl group transfer, is a key node in protecting cells against all these effects and has a significant impact on patient’s survival following temozolomide therapy, supporting the notion that findings obtained on a molecular and cellular level can be translated to the therapeutic setting in vivo. This comment summarizes the current knowledge on *O*^6^MeG-triggered pathways, including dose dependence and the question of thresholds, and comes up with the conclusion that data obtained on cell lines using low dose protocols are relevant and apoptosis, autophagy and senescence are therapeutically important endpoints.

In a recent review in *Biomedicines*, Strobel et al. discussed the mode of action of temozolomide, which is being used first-line in glioblastoma therapy [1]. Temozolomide (TMZ, Temodal^®^) is a spontaneously decomposing methylating agent, which acts similarly to dacarbazine (DTIC) that needs metabolic activation. TMZ was also frequently used in malignant melanoma therapy, but has been replaced in recent years by checkpoint inhibitors [2] and immunomodulators [3]. Although the authors provide a solid overview on the metabolism of methylating anticancer drugs, their reaction with the DNA, adduct formation and the role of membrane permeability, they largely neglected the cell biological effects of these S_N_1 methylating agents, for which a significant body of data is available (reviewed in [4,5]). Thus, they came to the conclusion that “…the only consistently shown effect of TMZ on cells is the increase of DNA content.” Furthermore, it was stated that “While TMZ has been shown to induce cell death, this is usually only produced in experimental systems with unphysiologically high concentrations, often in the range of 100 μM TMZ (ref) up to 1000 and 4000 μM (ref) while models predict a peak concentration in the tumor in the range of 14.95–34.54 μM (ref).” It was further concluded that “TMZ should be considered primarily cytostatic and senescence-inducing and not cytotoxic and apoptosis-inducing, potentially preventing cancer cells from G2 to M phase transition when tumor cells are most sensitive for mitotic cell death.” [1]. In my opinion, these are misleading and unsubstantiated statements that need comment.

First, there is solid data to show that TMZ and other S_N_1 methylating agents induce cell death by apoptosis [6,7], and the pathways activated by the critical lesion *O*^6^-methylguanine (*O*^6^MeG) are well described [4]. Thus, it has been shown that TMZ induces via processing of *O*^6^MeG/T mismatches DNA double-strand breaks (DSBs) in the post-treatment cell cycle [8,9] and triggers activation of the DNA damage kinases ATR and, as a secondary event, ATM [10], which in turn activate CHK1/CHK2-p53-driven apototic pathways [11]. It has also been shown that in p53 mutant glioma cells, the endogenous (mitochondrial) apoptosis pathway becomes activated, which is, however, less effective in triggering apoptosis than the exogenous p53 driven pathway [12]. Furthermore, it has been shown that TMZ induces autophagy and cellular senescence, which are important responses triggered by the *O*^6^MeG lesion [13,14]. It is important to note that these observed responses were evoked in glioblastoma cell lines with TMZ doses below and up to 50 µM [12,13].

In a recent study, the dose responses of these critical endpoints were assessed and it was shown that apoptosis, senescence (SA-βGal) and autophagy increased linearly with dose. Thus, even very low doses (between 5 and 25 µM), which are in the range of what is achieved systemically in the therapeutic setting [15,16,17], elicited toxic effects [18]. Interestingly, using the LN-229 and LN-308 (p53 wild-type) cell models, thresholds for apoptosis were not observed. This finding is compatible with the observation that the amount of γH2AX foci, which are an accepted marker of DSBs that trigger apoptosis, increases linearly with the dose of TMZ [18] and other *O*^6^-methylating agents (unpublished data). It should also be noted that *O*^6^MeG-derived secondary lesions activate upon treatment of glioblastoma cells with doses ≤50 µM TMZ the ATR/ATM-CHK1/2-SIAH1/HIPK2-p53Ser46 axis [19], which is considered to trigger the apoptosis pathway [20]. Furthermore, also the Jun kinase pathway, forcing both receptor-mediated and mitochondrial apoptosis via Fas ligand (*FASL*) and *BIM*, is activated following TMZ [21]. The pathways activated by *O*^6^MeG are outlined in Figure 1. Taken together, the available data show undoubtedly that the TMZ-induced DNA adduct *O*^6^MeG is a highly cytotoxic, genotoxic, recombinogenic and DNA damage response (DDR)-activating lesion. The data demonstrate at the same time that a single DNA repair protein, MGMT, is highly efficient in protecting against all these effects [22]. Importantly, already at low dose levels, *O*^6^MeG is a powerful activator of the apoptosis pathway [6,7], which can be explained by its MMR-mediated conversion into DSBs that trigger efficiently apoptosis without the involvement of PARP1 activation that stimulates necrosis [23].

Interestingly, *O*^6^MeG is also a powerful trigger of the senescence pathway [13]. Comparing the responses under identical treatment conditions, we observed with a dose of 15 µM TMZ yields of about 20% apoptosis (annexin V positive) and 42% senescence (C_12_FDG positive) in LN-229 glioblastoma cells [18]. Thus, it appears that senescence is a major pathway activated by *O*^6^MeG lesions, which does not mean that apoptosis is not induced and irrelevant for explaining the cytotoxic effects. Of note, we do not know whether senescent cells die by apoptosis or by another process at a later stage. In view of the bulk of published data (for an extended list of references see [5]) the statement that “TMZ should be considered primarily cytostatic and senescence-inducing and not cytotoxic and apoptosis-inducing” is not tenable.

I do agree with the authors that, in some publications, unusually high concentrations of TMZ were used, which are even in the mM range. These high doses are required to elicit cytotoxic and genotoxic effects in MGMT expressing, TMZ-resistant cells and are brought about by N-alkylations and other adducts repaired by base excision repair (BER) and ALKBH2 [5]. At these high dose levels, the cellular BER and ALKBH2 repair capacity appears to be saturated and, therefore, these adducts become the preponderant toxic insults. It is true that these effects elicited at very high doses of TMZ cannot be achieved in vivo, unless the tumor is impaired in BER and/or ALKBH2. Therefore, responses observed in MGMT expressing (MGMT promoter unmethylated) cell models and high TMZ doses (>100 µM) should be taken with caution.

It should also be taken into consideration that in the therapeutic setting TMZ is administered repeatedly with daily doses of 50 to 130 mg/m^2^ or even higher [24,25]. Under these conditions, a huge accumulation of *O*^6^MeG in the DNA is expected to occur in MGMT lacking (MGMT promoter methylation positive) tumors, strongly enhancing the DNA damage response, signal activation and activation of apoptosis and senescence pathways. Therefore, the biologically effective dose in a therapeutic setting is even likely higher than what is achieved in vitro in most of the experimental settings, when single doses of up to 100 µM are used.

TMZ is clearly a cytotoxic drug, as cells following treatment die by apoptosis even at low dose levels [18,19]. Since TMZ induces, like all genotoxic agents, DNA synthesis inhibition and cell cycle arrest as well as cellular senescence, it may also be considered a cytostatic drug (i.e., cells are just inhibited in proliferation). However, measuring these endpoints, it remains unclear whether cells blocked in particular cell cycle positions or the senescent state are irreversibly arrested or only transiently, and finally enter a death pathway (or continue to proliferate). S-phase inhibition and cell cycle delay following DNA damage are well-known transient phenomena, which should not be considered as a cytostatic activity. It should also be noted that cell cycle inhibition and senescence are regulated, at least in part, by the same upstream DNA damage response players that regulate apoptosis. For these reasons, TMZ should be considered a cytotoxic agent rather than a cytostatic drug.

To the best of my knowledge, neither apoptosis nor senescence has been demonstrated in human glioblastoma specimens obtained after resection upon the first therapy cycle. However, this lack of data should not be taken as an argument that glioblastoma cells do not die by apoptosis following therapy. If apoptotic markers are not detectable following therapy, this may result from the clearance of apoptotic cells in the recurrent tumor in the period between the end of TMZ treatment and resection. The important role of MGMT, which was first discovered in bacteria [26], intensively assessed as a key defense [27] and drug resistance mechanism in cancer cell lines in vitro [28,29] and then translated to the tumor response [30], is an impressive example for demonstrating that processes occurring on a molecular level can be translated to cancer cells that grow in a much more complex tumor environment.

## Figures and Tables

**Figure 1:**
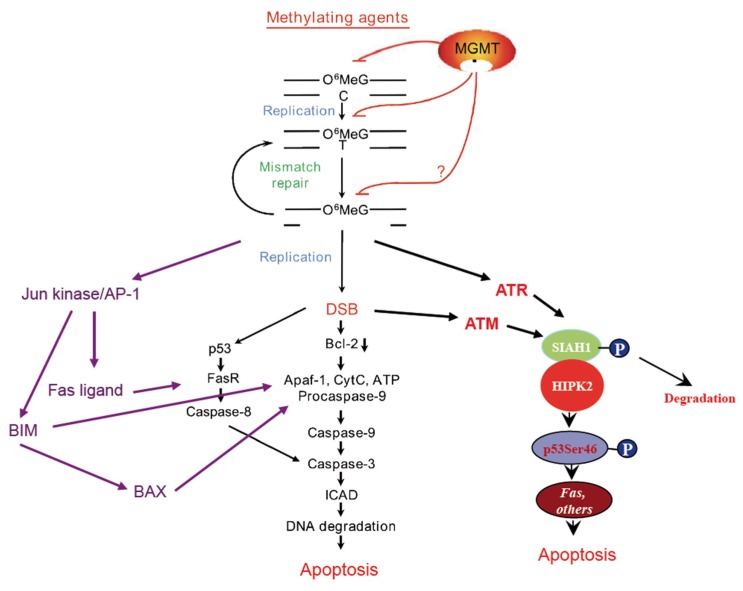
Cell death pathways triggered by the temozolomide-induced DNA lesion *O*^6^-methylguanine. Although TMZ, like other S_N_1 alkylators, induces more than a dozen DNA adducts, *O*^6^MeG is the key cytotoxic lesion and MGMT the primary node of drug resistance (reviewed in Reference [22]). The damage is converted into DSBs and, in p53 mutated cells, stimulates mitochondrial apoptosis, a hallmark of which is Bcl-2 decline [8]. In p53 wild-type cells, apoptosis is additionally driven by death receptor triggered caspase-8 activation, which requires upregulation of the Fas receptor controlled by p53 [12], and the Fas ligand, which is under control of AP-1. Additionally, the AP-1 dependent BIM/BAX apoptosis pathway [21], as well as the SIAH1/HIPK2-p53Ser46 pathway become activated [19] thus contributing to cell death executed by apoptosis. The model is based on experiments with TMZ doses of ≤50 µM, which were used for glioblastoma cell treatment in the cited works.
